# 689. Pediatric vs. Adult Invasive Aspergillosis in Cancer and Hematopoietic Stem Cell Transplant Patients: Insights from a Matched Cohort at a Tertiary Cancer Center

**DOI:** 10.1093/ofid/ofaf695.228

**Published:** 2026-01-11

**Authors:** Saliba Wehbe, ramia g zakhour, Ray Y Hachem, Hiba Dagher, Ying Jiang, Anne-Marie Chaftari, Issam I Raad

**Affiliations:** The University of Texas, MD Anderson Cancer Center, Houston, TX; University of Texas at Houston, houston, Texas; MD Anderson UT, Houston, Texas; UT MD Anderson Cancer Center, Houston, Texas; The University of Texas MD Anderson Cancer Center, Houston, Texas; MD Anderson UT, Houston, Texas; MD Anderson UT, Houston, Texas

## Abstract

**Background:**

Invasive aspergillosis (IA) remains the most common invasive mold infection in pediatric and adult patients with cancer or hematopoietic stem cell transplant (HCT), leading to substantial morbidity and mortality. Differences in IA presentation, diagnosis, management, and outcomes between children and adults have been proposed, but most clinical guidelines are based on adult data. Generating pediatric-specific evidence and conducting direct comparisons with adult IA are critical to informing tailored strategies for children.

**Figure:**
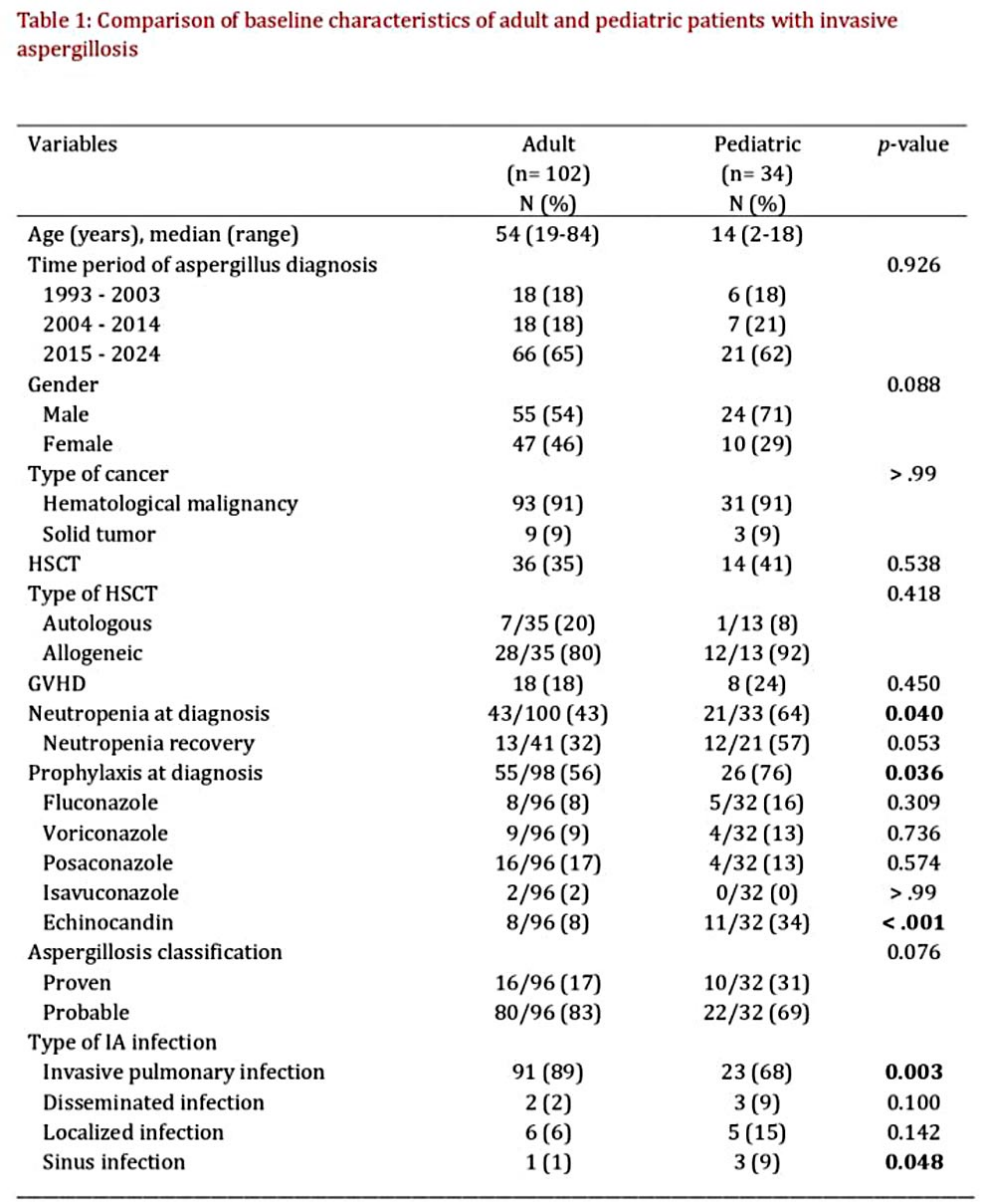

**Figure:**
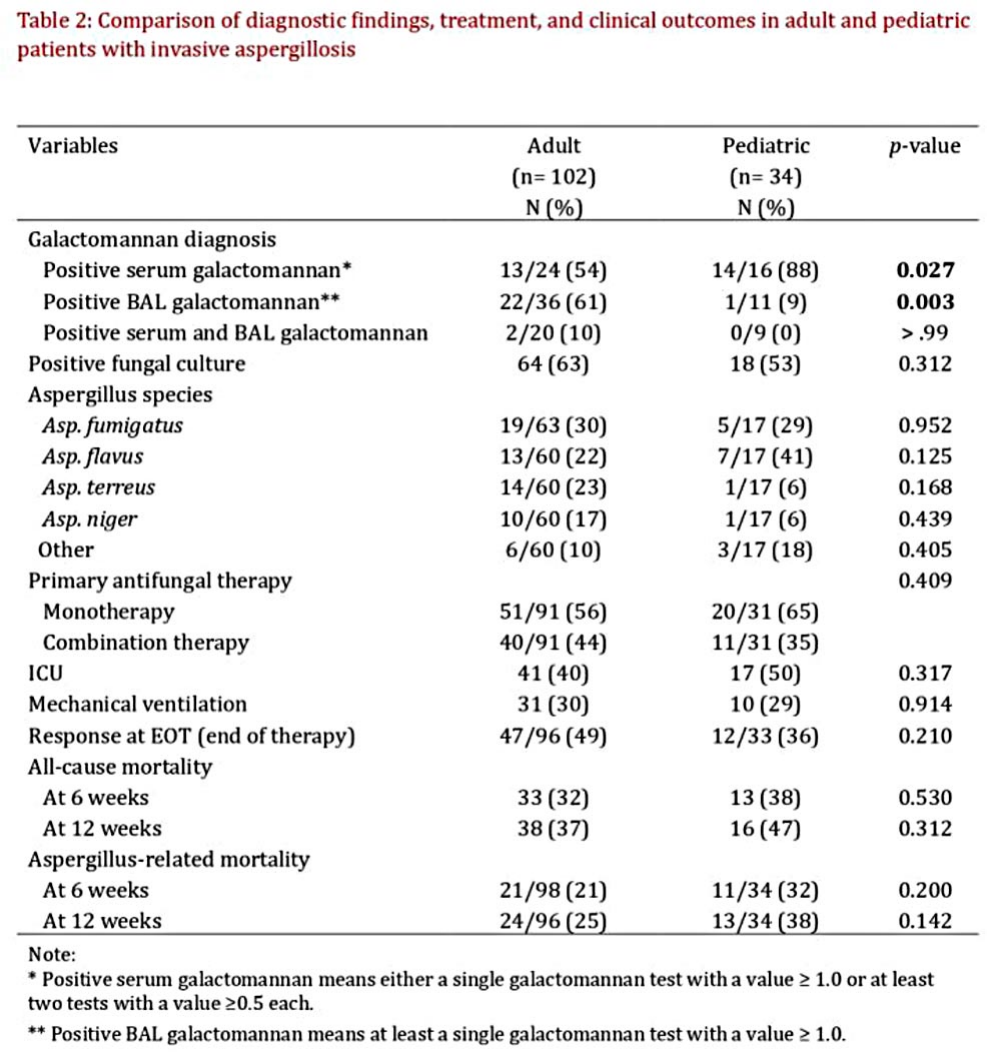

**Methods:**

We retrospectively reviewed proven and probable IA cases diagnosed at MD Anderson Cancer Center from 1993 to 2024. 34 pediatric patients (≤18 years) were matched 1:3 with 102 adult patients by diagnosis year (±1), cancer type (hematologic malignancy vs. solid tumor), and history of HCT within one year. Adults were selected using simple random sampling.

**Results:**

Neutropenia at diagnosis was more frequent among children (p= 0.04). Patterns of antifungal prophylaxis differed notably, with less children receiving a mold-active azole at the time of IA, and a higher percentage receiving an echinocandin (34% vs 8% for adults, p< 0.001). Extrapulmonary IA was more common in children, with 89% of adults having pulmonary IA vs. 68% of children (p=0.003). Serum galactomannan was more commonly positive in children (p=0.027), while bronchoalveolar lavage (BAL) galactomannan was more frequently positive in adults (p=0.003). Among culture-confirmed cases, *A. flavus* was most commonly isolated in children, followed by *A. fumigatus*, though species distribution overall did not differ significantly. Pediatric patients were more often treated with monotherapy. When it comes to outcomes, ICU admission and mechanical ventilation rates were comparable between groups. At 12 weeks, both all-cause (47% vs. 37%) and IA-attributable mortality (38% vs. 25%) were higher in the pediatric group, though these differences did not reach statistical significance.

**Conclusion:**

IA continues to cause high morbidity and mortality in pediatric cancer and HCT patients. Our findings reveal distinct clinical, diagnostic, and treatment patterns in children compared to adults, underscoring the need for pediatric-specific IA management guidelines.

**Disclosures:**

All Authors: No reported disclosures

